# Supplementary material for: Disruptor: Computational identification of oncogenic mutants disrupting protein-protein and protein-DNA interactions
Source: Commun Biol. 2023 Jul 13;6:720. doi: 10.1038/s42003-023-05089-2 (PMC10344873; doi:10.1038/s42003-023-05089-2)
Supplement: Supplementary file 1 — Supplementary_Information [file 42003_2023_5089_MOESM1_ESM.pdf]

Supplementary information

Disruptor: Computational identification of oncogenic mutants disrupting protein-protein and protein-DNA interactions

Valentina Kugler,[1] Andreas Lieb,[2] Nathan Guerin,[3] Bruce R. Donald,[3,4,5,6] Eduard Stefan,[1,7,8] Teresa Kaserer[9]*

[1] Institute of Biochemistry and Center for Molecular Biosciences, University of Innsbruck, Innsbruck, Austria

[2] Institute of Pharmacology, Medical University of Innsbruck, Innsbruck, Austria

[3] Department of Computer Science, Duke University, Durham, USA

[4] Department of Biochemistry, Duke University Medical Center, Durham, USA

[5] Department of Chemistry, Duke University, Durham, USA

[6] Department of Mathematics, Duke University, Durham, USA

[7] Tyrolean Cancer Research Institute (TKFI), Innrain 66, 6020 Innsbruck, Austria.

[8] Institute of Molecular Biology, University of Innsbruck, Innsbruck, Austria

[9] Institute of Pharmacy / Pharmaceutical Chemistry, University of Innsbruck, Innsbruck, Austria

* Corresponding author: teresa.kaserer@uibk.ac.at

Supplementary Table 1. Prioritized hotspot mutations for p53 chain A predicted to disrupt interactions DNA consensus sequence.

wt residue[1]	mutant	K*(Log$_{10}$)	ΔK* (Log$_{10}$) score[2]	COSMIC[3]
K101	A	2,34299	-3,852917	-
K101	N	2,383493	-3,812414	1
K101	D	-0,10607	-6,301977	-
K101	Q	2,541033	-3,654874	-
K101	E	-0,281937	-6,477844	-
K101	G	2,329185	-3,866722	-
K101	H	2,408996	-3,786911	-
K101	I	2,385238	-3,810669	-
K101	L	2,422414	-3,773493	-
K101	M	2,432355	-3,763552	-
K101	P	2,347536	-3,848371	-
K101	S	2,365646	-3,830261	-
K101	T	2,40944	-3,786467	-
K101	Y	2,590051	-3,605856	-
K101	V	2,376722	-3,819185	-
R249	A	0,517047	-3,879753	-
R249	N	0,321083	-4,075717	-
R249	C	0,476555	-3,920245	-
R249	Q	1,173259	-3,223541	-
R249	E	-3,828045	-8,224845	-
R249	G	0,477414	-3,919386	68
R249	I	0,674289	-3,722511	1
R249	L	0,816013	-3,580787	-
R249	M	0,707806	-3,688994	78
R249	P	none	X	-
R249	S	0,573587	-3,823213	609
R249	T	0,448275	-3,948525	5046
R249	W	1,102095	-3,294705	61
R249	V	0,629113	-3,767687	1
R248	A	0,203102	-4,698122	-
R248	C	0,321923	-4,579301	1
R248	Q	0,431464	-4,46976	1520
R248	E	-3,03584	-7,937064	-
R248	G	0,181438	-4,719786	36
R248	H	0,37264	-4,528584	1
R248	I	0,288149	-4,613075	-
R248	M	0,307344	-4,59388	-

R248	P	none	X	40
R248	S	0,298687	-4,602537	-
R248	T	0,077249	-4,823975	-
R248	W	0,351411	-4,549813	1193
R248	V	0,265969	-4,635255	-

[1] wildtype residue, [2]difference of K* score of mutant in relation to wt residue, [3] number of patient samples harboring the mutant reported in COSMIC, none… no binding at all was predicted for the mutant, X… thus no difference of the K* score to the wt could be calculated.

Supplementary Table 2. Prioritized hotspot mutations for p53 chain B predicted to disrupt interactions DNA consensus sequence.

wt residue[1]	mutant	K*(Log_{10})	ΔK* (Log_{10}) score[2]	COSMIC[3]
K120	A	2,691146	-11,188792	-
K120	N	4,108135	-9,771803	3
K120	D	-4,373352	-18,25329	-
K120	Q	5,186083	-8,693855	1
K120	E	-4,313321	-18,193259	21
K120	G	1,991899	-11,888039	-
K120	H	-10,93979	-24,819728	-
K120	I	-7,360472	-21,24041	-
K120	L	4,388371	-9,491567	-
K120	M	4,931815	-8,948123	10
K120	P	none	X	-
K120	S	2,930995	-10,948943	-
K120	T	3,46453	-10,415408	3
K120	W	none	X	-
K120	Y	none	X	-
K120	V	4,020949	-9,858989	-
R273	A	10,016722	-7,825299	-
R273	N	10,008983	-7,833038	-
R273	D	5,055741	-12,78628	-
R273	C	9,978956	-7,863065	1379
R273	Q	10,768056	-7,073965	2
R273	G	9,989578	-7,852443	27
R273	H	10,095936	-7,746085	1406
R273	I	10,197308	-7,644713	-
R273	L	10,283987	-7,558034	278
R273	F	11,116865	-6,725156	-
R273	P	none	X	56
R273	S	9,967608	-7,874413	43
R273	T	10,105834	-7,736187	-
R273	W	11,407796	-6,434225	-
R273	Y	11,329325	-6,512696	1
R273	V	10,108209	-7,733812	-
R283	A	0,606024	-4,514618	-
R283	N	0,89169	-4,228952	-
R283	D	-2,922849	-8,043491	-
R283	C	0,706364	-4,414278	37
R283	Q	0,555647	-4,564995	-

R283	G	0,590585	-4,530057	3
R283	H	0,742676	-4,377966	23
R283	I	0,695116	-4,425526	-
R283	L	0,669899	-4,450743	4
R283	F	0,658977	-4,461665	-
R283	P	none	X	56
R283	S	0,630511	-4,490131	1
R283	T	0,638687	-4,481955	-
R283	W	0,647427	-4,473215	-
R283	Y	0,670915	-4,449727	-
R283	V	0,65277	-4,467872	-

[1] wildtype residue, [2]difference of K* score of mutant in relation to wt residue, [3] number of patient samples harboring the mutant reported in COSMIC, none… no binding at all was predicted for the mutant, X… thus no difference of the K* score to the wt could be calculated.

Supplementary Table 3. Prioritized hotspot mutations for p53 chain C predicted to disrupt interactions DNA consensus sequence.

wt residue[1]	mutant	K*(Log_{10})	ΔK* (Log_{10}) score[2]	COSMIC[3]
K120	A	1,815678	-6,933418	-
K120	N	2,775805	-5,973291	3
K120	D	-3,462315	-12,211411	-
K120	Q	2,493117	-6,255979	1
K120	E	-3,730755	-12,479851	21
K120	G	1,873719	-6,875377	-
K120	H	3,06706	-5,682036	-
K120	I	-39,45776	-48,206856	-
K120	L	2,954455	-5,794641	-
K120	M	3,706782	-5,042314	10
K120	P	2,544973	-6,204123	-
K120	S	2,16749	-6,581606	-
K120	T	-19,04392	-27,793016	3
K120	W	4,1289	-4,620196	-
K120	Y	3,522574	-5,226522	-
K120	V	-56,05519	-64,804286	-
R280	A	-2,73018	-4,932897	-
R280	N	-2,896371	-5,099088	-
R280	C	-2,602158	-4,804875	-
R280	Q	-2,396227	-4,598944	-
R280	E	-7,085311	-9,288028	-
R280	G	-2,799111	-5,001828	78
R280	I	-2,419549	-4,622266	50
R280	L	-2,313941	-4,516658	-
R280	M	-2,239468	-4,442185	-
R280	P	-2,619168	-4,821885	-
R280	S	-2,751042	-4,953759	39
R280	T	-2,635841	-4,838558	174
R280	W	-1,659759	-3,862476	-
R280	V	-2,540043	-4,74276	-
R248	A	1,724592	-3,233005	-
R248	C	1,856652	-3,100945	1
R248	Q	1,727043	-3,230554	1520
R248	E	-1,084808	-6,042405	-
R248	G	1,556304	-3,401293	36
R248	H	1,888048	-3,069549	1
R248	L	-2,518818	-7,476415	159

R248	P	none	X	40
R248	S	1,737028	-3,220569	-
R248	W	-0,814039	-5,771636	1193

[1] wildtype residue, [2]difference of K* score of mutant in relation to wt residue, [3] number of patient samples harboring the mutant reported in COSMIC, none… no binding at all was predicted for the mutant, X… thus no difference of the K* score to the wt could be calculated.

Supplementary Table 4. Prioritized hotspot mutations for p53 predicted to disrupt interactions with ASPP2. Please note that four residue positions are reported, because an equal number of individual mutations was predicted for residues 248,249, 280.

wt residue[1]	mutant	K*(Log_{10})	ΔK* (Log_{10}) score[2]	COSMIC[3]
R273	A	0,323495	-4,932792	-
R273	N	0,269833	-4,986454	-
R273	D	-2,454937	-7,711224	-
R273	C	0,277161	-4,979126	1379
R273	Q	0,661741	-4,594546	2
R273	G	0,309248	-4,947039	27
R273	H	0,239307	-5,01698	1406
R273	I	0,346589	-4,909698	-
R273	L	0,363471	-4,892816	278
R273	F	0,560962	-4,695325	-
R273	P	none	X	56
R273	S	0,295117	-4,96117	43
R273	T	0,351097	-4,90519	-
R273	W	0,891786	-4,364501	-
R273	Y	0,535113	-4,721174	1
R273	V	0,343263	-4,913024	-
R248	A	5,193638	-4,511186	-
R248	C	6,215555	-13,489269	1
R248	Q	3,323453	-16,381371	1520
R248	E	-3,524651	-23,229475	-
R248	G	5,141793	-14,563031	36
R248	H	3,29916	-16,405664	1
R248	L	3,921772	-15,783052	159
R248	K	14,533442	-5,171382	-
R248	M	7,055204	-12,64962	-
R248	P	none	X	40
R248	S	5,490434	-14,21439	-
R248	T	-15,45115	-35,155974	-
R248	W	-33,78764	-53,492464	1193
R248	V	6,466922	-13,237902	-
R249	A	15,437851	-4,266973	-
R249	N	14,964978	-4,739846	-
R249	C	15,689048	-4,015776	-
R249	Q	13,903387	-5,801437	-
R249	E	10,255063	-9,449761	-
R249	G	15,266362	-4,438462	68

R249	I	16,043865	-3,660959	1
R249	L	6,003125	-13,701699	-
R249	M	16,406264	-3,29856	78
R249	P	none	X	-
R249	S	15,557841	-4,146983	609
R249	T	15,519187	-4,185637	50
R249	W	none	X	61
R249	V	16,015213	-3,689611	1
R280	A	0,475741	-5,638805	-
R280	N	0,825332	-5,289214	-
R280	C	0,533749	-5,580797	-
R280	Q	0,634461	-5,480085	-
R280	E	-1,778061	-7,892607	-
R280	G	0,453977	-5,660569	78
R280	I	0,56953	-5,545016	50
R280	L	0,645035	-5,469511	-
R280	M	0,837309	-5,277237	-
R280	P	none	X	-
R280	S	0,539367	-5,575179	39
R280	T	0,58243	-5,532116	174
R280	W	1,116796	-4,99775	-
R280	V	0,546488	-5,568058	-

[1] wildtype residue, [2]difference of K* score of mutant in relation to wt residue, [3] number of patient samples harboring the mutant reported in COSMIC, none… no binding at all was predicted for the mutant, X… thus no difference of the K* score to the wt could be calculated.

Supplementary Table 5. Prioritized hotspot mutations for ERK2 predicted to disrupt interactions with DUSP6.

wt residue[1]	mutant	K*(Log$_{10}$)	ΔK* (Log$_{10}$) score[2]	Pb[3] colorectal cancer	Pb[3] cervical cancer	COSMIC[4]
D321	N	-1,182303	-8,764402	0,00402124	0,00646836	8
D321	H	-14,64852	-22,230619	0,00096405	0,00477121	-
D321	Y	None[5]	X[6]	0,00128344	0,00352652	1
D321	G	0,123075	-7,459024	0,00604358	0,00298779	2
D321	V	0,346748	-7,235351	0,00064382	0,00246853	1
D321	A	0,272173	-7,309926	0,00091117	0,00146627	1
D321	E	-4,191007	-11,773106	0,00042221	0,00142358	1
D321	S	-0,208939	-7,791038	2,4673E-05	2,3709E-05	-
D321	I	-3,383509	-10,965608	2,5077E-06	1,5454E-05	-
D321	R	-12,0264	-19,608499	5,6435E-06	1,3797E-05	-
D321	L	-16,51716	-24,099259	6,012E-07	1,1399E-05	-
D321	C	0,639544	-6,942555	7,5132E-06	1,0198E-05	-
D321	T	-3,179452	-10,761551	3,5491E-06	9,1792E-06	-
D321	K	-9,397628	-16,979727	1,6713E-06	9,0652E-06	-
D321	F	none	X	8,0038E-07	8,4253E-06	-
D321	P	none	X	8,5085E-07	6,7708E-06	-
D321	Q	-4,940341	-12,52244	4,0068E-07	6,6867E-06	-
D318	N	-0,212286	-4,309833	0,00999477	0,02316461	1
D318	H	0,930958	-3,166589	0,00011049	0,00341755	-
D318	G	0,648646	-3,448901	0,00457925	0,00240737	-
D318	Y	0,909629	-3,187918	0,00067654	0,00231833	-
D318	V	0,788937	-3,30861	0,00065276	0,00130214	-
D318	A	0,693979	-3,403568	0,00025986	0,00047436	-
D318	K	-1,872717	-5,970264	2,3788E-05	0,00018536	-
D318	S	0,647953	-3,449594	4,5487E-05	5,5821E-05	-
D318	I	-0,010491	-4,108038	6,4592E-06	0,00002961	-
D318	Q	0,52897	-3,568577	2,6297E-07	2,7347E-05	-
D318	T	0,960081	-3,137466	2,5714E-06	1,0787E-05	-
D318	R	-2,143087	-6,240634	5,0092E-07	8,0762E-06	-
D318	C	0,418232	-3,679315	3,0672E-06	5,4786E-06	-
D318	L	-2,06998	-6,167527	7,1404E-08	4,3684E-06	-
D318	F	1,005684	-3,091863	4,3722E-07	2,9633E-06	-
D318	P	none	X	2,8426E-08	1,5914E-06	-
E81	K	-0,356733	-5,721186	0,00397595	0,00679256	5
E81	Q	2,323264	-3,041189	0,00095319	0,00501034	-
E81	G	1,556504	-3,807949	0,00561014	0,0035114	-

E81	V	1,735473	-3,62898	0,00093775	0,00209152	-
E81	A	1,59246	-3,771993	0,00070536	0,001344	-
E81	N	1,803066	-3,561387	2,7476E-05	0,000647	-
E81	H	2,091465	-3,272988	6,5871E-06	0,00047724	-
E81	Y	2,151015	-3,213438	8,7695E-06	0,00035274	-
E81	R	0,386516	-4,977937	2,7223E-05	3,3257E-05	-
E81	L	2,071127	-3,293326	2,0514E-06	1,4624E-05	-
E81	W	2,223596	-3,140857	7,0083E-06	1,0435E-05	-
E81	T	1,724047	-3,640406	2,7608E-06	7,3257E-06	-
E81	P	1,603678	-3,760775	6,6187E-07	5,4036E-06	-
E81	S	1,64994	-3,714513	8,8115E-07	3,9939E-06	-

[1] wildtype residue, [2]difference of K* score of mutant in relation to wt residue, [3] Pb relative Probability for a mutation to be formed in the indicated cancer type, [4] number of patient samples harboring the mutant reported in COSMIC, none… no binding at all was predicted for the mutant, X… thus no difference of the K* score to the wt could be calculated.

Supplementary Table 6. Prioritized hotspot mutations for p16 predicted to disrupt interactions with CDK6. Please note that four residue positions are reported, because an equal number of individual mutations was predicted for residues G23, G55, G89.

wt residue[1]	mutant	K*(Log$_{10}$)	ΔK* (Log$_{10}$) score[2]	Pb[3] melanoma	COSMIC[4]
D84	A	0,705794	-4,822505	5,1003E-05	1
D84	R	none	X	4,9293E-05	-
D84	N	0,267755	-5,260544	0,07688685	37
D84	C	1,413534	-4,114765	2,2295E-07	-
D84	Q	-12,0721	-17,600399	4,3045E-08	-
D84	E	-9,249456	-14,777755	0,00015851	-
D84	G	0,330964	-5,197335	0,00064395	15
D84	H	none	X	0,00027263	2
D84	I	none	X	1,3085E-05	-
D84	L	-57,34838	-62,876679	4,6397E-08	-
D84	K	-39,35921	-44,887509	1,214E-05	-
D84	F	none	X	5,9392E-08	-
D84	P	none	X	1,3794E-08	-
D84	S	1,151568	-4,376731	4,9137E-05	-
D84	T	1,457733	-4,070566	3,8903E-06	-
D84	Y	none	X	0,00034898	33
D84	V	none	X	0,00017154	6
G23	A	none	X	0,00024447	-
G23	R	none	X	0,00049361	2
G23	N	none	X	0,00125316	-
G23	D	none	X	0,01622997	2
G23	C	none	X	0,00073695	1
G23	E	none	X	3,595E-06	-
G23	H	none	X	7,7217E-06	-
G23	I	none	X	6,9919E-05	-
G23	L	none	X	4,3083E-07	-
G23	F	none	X	6,6648E-07	-
G23	P	none	X	1,1628E-07	-
G23	S	none	X	0,07731202	6
G23	T	none	X	1,8872E-05	-
G23	W	none	X	3,741E-08	-
G23	Y	none	X	1,1945E-05	-
G23	V	none	X	0,00090574	5
G55	A	none	X	6,096E-07	-
G55	R	none	X	0,00057867	3
G55	N	none	X	0,00136281	-

G55	D	none	X	0,01766393	2
G55	C	none	X	0,00073753	1
G55	E	none	X	2,3125E-05	-
G55	H	none	X	8,3974E-06	-
G55	I	none	X	3,5426E-05	-
G55	L	none	X	2,1829E-07	-
G55	F	none	X	3,3769E-07	-
G55	P	none	X	2,2125E-07	-
G55	S	none	X	0,07737281	-
G55	T	none	X	3,5907E-05	-
G55	W	none	X	9,5684E-08	-
G55	Y	none	X	1,2991E-05	-
G55	V	none	X	0,00045977	3
G89	A	none	X	0,00047142	-
G89	R	none	X	0,00053999	-
G89	N	none	X	0,00136281	-
G89	D	none	X	0,01787906	1
G89	C	none	X	0,00074651	2
G89	E	none	X	1,3031E-05	-
G89	H	none	X	8,3974E-06	-
G89	I	none	X	3,5426E-05	-
G89	L	none	X	2,1829E-07	-
G89	F	none	X	3,3769E-07	-
G89	P	none	X	2,2125E-07	-
G89	S	none	X	0,07831512	6
G89	T	none	X	3,5907E-05	-
G89	W	none	X	1,4967E-07	-
G89	Y	none	X	1,2991E-05	-
G89	V	none	X	0,0004651	2

[1] wildtype residue, [2]difference of K* score of mutant in relation to wt residue, [3] Pb relative Probability for a mutation to be formed in the indicated cancer type, [4] number of patient samples harboring the mutant reported in COSMIC, none… no binding at all was predicted for the mutant, X… thus no difference of the K* score to the wt could be calculated.

Supplementary Table 7. Prioritized hotspot mutations for CDK6 predicted to disrupt interactions with p16. Please note that four residue positions are reported, because an equal number of individual mutations was predicted for residues G37 and R31, G20 and K111.

wt residue[1]	mutant	K*(Log$_{10}$)	ΔK* (Log$_{10}$) score[2]	COSMIC[3]
G37	A	none	X	
G37	R	none	X	
G37	N	none	X	
G37	D	none	X	
G37	C	none	X	
G37	E	none	X	
G37	H	none	X	
G37	I	none	X	
G37	L	none	X	
G37	F	none	X	
G37	P	none	X	
G37	S	none	X	
G37	T	none	X	
G37	W	none	X	
G37	Y	none	X	
G37	V	none	X	
R31	A	0.798177	-5.643494	
R31	N	1.248857	-5.192814	
R31	D	-1.09348	-7.535152	
R31	C	1.339103	-5.102568	
R31	Q	0.164235	-6.277436	
R31	G	0.394287	-6.047384	
R31	H	2.442278	-3.999393	
R31	I	none	X	
R31	L	-3.61789	-10.059558	
R31	F	none	X	
R31	P	none	X	
R31	S	0.799319	-5.642352	
R31	T	1.403068	-5.038603	
R31	W	none	X	
R31	Y	none	X	
R31	V	1.606236	-4.835435	
G20	A	none	X	
G20	R	none	X	
G20	D	none	X	
G20	C	none	X	

G20	Q	none	X	
G20	E	none	X	
G20	L	none	X	
G20	K	none	X	
G20	M	none	X	
G20	P	none	X	
G20	S	none	X	
G20	T	none	X	
G20	W	none	X	
G20	V	none	X	
K111	A	1.493153	-4.234931	
K111	D	-0.173548	-5.901632	
K111	Q	2.712797	-3.015287	
K111	E	0.421897	-5.306187	
K111	G	1.310223	-4.417861	
K111	H	-66.34503	-72.073114	
K111	I	-10.72942	-16.457504	
K111	L	1.606365	-4.121719	
K111	M	1.894562	-3.833522	
K111	P	none	X	
K111	S	1.558725	-4.169359	
K111	T	1.949978	-3.778106	
K111	Y	none	X	
K111	V	2.363409	-3.364675	

[1] wildtype residue, [2]difference of K* score of mutant in relation to wt residue, [3] number of patient samples harboring the mutant reported in COSMIC, none… no binding at all was predicted for the mutant, X… thus no difference of the K* score to the wt could be calculated.

Supplementary Table 8. Prioritized hotspot mutations for smad4 predicted to disrupt interactions with smad2. Please note that five residue positions are reported, because an equal number of individual mutations was predicted for residues 361, 365, 428, 507.

wt residue[1]	mutant	K*(Log10)	ΔK* (Log10) score[2]	Pb[3] colorectal	COSMIC[4]
D537	A	4.582058	-4.821045	0.00026246	4
D537	R	none	X	5.2273E-06	
D537	N	4.470593	-4.93251	0.00448332	
D537	C	4.884818	-4.518285	2.6362E-05	
D537	Q	-24.48158	-33.884683	4.8495E-07	
D537	E	2.371261	-7.031842	0.00042221	7
D537	G	3.761375	-5.641728	0.00462508	20
D537	H	none	X	0.00116682	6
D537	I	none	X	2.8631E-06	
D537	L	none	X	7.4514E-07	
D537	K	none	X	1.8634E-06	
D537	F	none	X	3.7578E-06	
D537	P	none	X	2.9663E-07	
D537	S	4.739755	-4.663348	2.1581E-05	
D537	T	2.886855	-6.516248	1.1398E-06	
D537	Y	none	X	0.00588437	29
D537	V	-19.50471	-28.907813	0.00065929	14
R361	A	0.974539	-4.772984	2.8985E-09	
R361	N	1.028212	-4.719311	1.118E-05	
R361	D	0.266894	-5.480629	1.8259E-06	
R361	C	1.065402	-4.682121	0.01002855	167
R361	Q	0.886778	-4.860745	4.1914E-05	
R361	G	0.959733	-4.78779	0.00011114	15
R361	H	1.062984	-4.684539	0.01670801	210
R361	I	1.070326	-4.677197	2.5094E-07	
R361	L	1.089429	-4.658094	0.00037596	4
R361	F	1.023549	-4.723974	2.659E-05	
R361	P	none	X	0.00067909	3
R361	S	0.976897	-4.770626	0.00067909	14
R361	T	0.988378	-4.759145	1.7748E-08	
R361	W	1.545709	-4.201814	6.2797E-06	
R361	Y	1.015874	-4.731649	0.00016517	
R361	V	1.025202	-4.722321	4.0982E-08	
G365	A	none	X	0.00073185	1
G365	R	none	X	0.00042811	1
G365	N	none	X	1.4458E-05	

G365	D	none	X	0.00383563	5
G365	C	none	X	0.00274886	
G365	E	none	X	3.4631E-06	
G365	H	none	X	1.6127E-06	
G365	I	none	X	6.2906E-06	
G365	L	none	X	7.0167E-07	
G365	F	none	X	4.5458E-06	
G365	P	none	X	3.0744E-07	
G365	S	none	X	0.0038059	4
G365	T	none	X	2.7562E-06	
G365	W	none	X	7.0669E-07	
G365	Y	none	X	1.0448E-05	
G365	V	none	X	0.00167031	2
K428	A	0.126469	-6.547002	3.6755E-06	
K428	N	0.448996	-6.224475	0.00694114	
K428	D	-1.760524	-8.433995	1.4012E-05	
K428	Q	0.903522	-5.769949	0.00026279	
K428	E	-2.886629	-9.5601	0.00202761	
K428	G	0.102477	-6.570994	1.3902E-05	
K428	H	0.183194	-6.490277	1.8161E-06	
K428	I	0.333433	-6.340038	1.1911E-05	
K428	L	0.411409	-6.262062	2.7446E-07	
K428	M	0.175197	-6.498274	0.00104902	
K428	P	0.158751	-6.51472	4.7637E-07	
K428	S	0.14608	-6.527391	4.927E-05	
K428	T	0.184828	-6.488643	0.00184139	
K428	W	0.119359	-6.554112	5.5573E-06	
K428	Y	0.147132	-6.526339	5.6013E-06	
K428	V	0.196069	-6.477402	2.1177E-06	
K507	A	2.539634	-8.242713	9.6383E-06	
K507	R	5.220245	-5.562102	0.00391035	
K507	N	2.963475	-7.818872	0.00286974	4
K507	D	-1.696103	-12.47845	1.2285E-05	
K507	Q	0.219739	-10.562608	0.00073351	3
K507	E	-6.18591	-16.968257	0.00431049	5
K507	G	2.343533	-8.438814	1.6626E-05	
K507	H	3.447321	-7.335026	2.0906E-06	
K507	I	none	X	0.00096382	
K507	L	none	X	1.6549E-06	
K507	M	3.926115	-6.856232	6.6185E-06	
K507	P	none	X	1.6401E-06	
K507	S	2.548982	-8.233365	1.3382E-05	

K507	T	2.970434	-7.811913	0.00227337	
K507	Y	4.805533	-5.976814	2.8508E-06	
K507	V	none	X	4.1143E-06	

[1] wildtype residue, [2]difference of K* score of mutant in relation to wt residue, [3] Pb relative Probability for a mutation to be formed in the indicated cancer type, [4] number of patient samples harboring the mutant reported in COSMIC, none… no binding at all was predicted for the mutant, X… thus no difference of the K* score to the wt could be calculated.

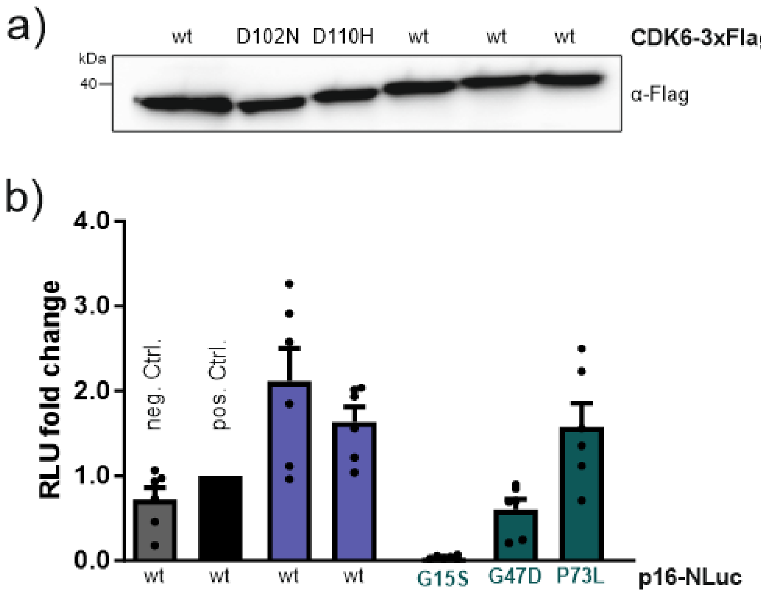

Supplementary Figure 1. a) Immunoblotting shows expression of Flag tagged CDK6 variants. **b)** Bioluminescence signals of 1% input samples of LUMIER experiments. Bars represent the luciferase intensities of p16-NLuc variants relative to the wild-type. Input signals were used for normalization of signals after immunoprecipitation. Error bars represent SEM with n = 6 experiments.

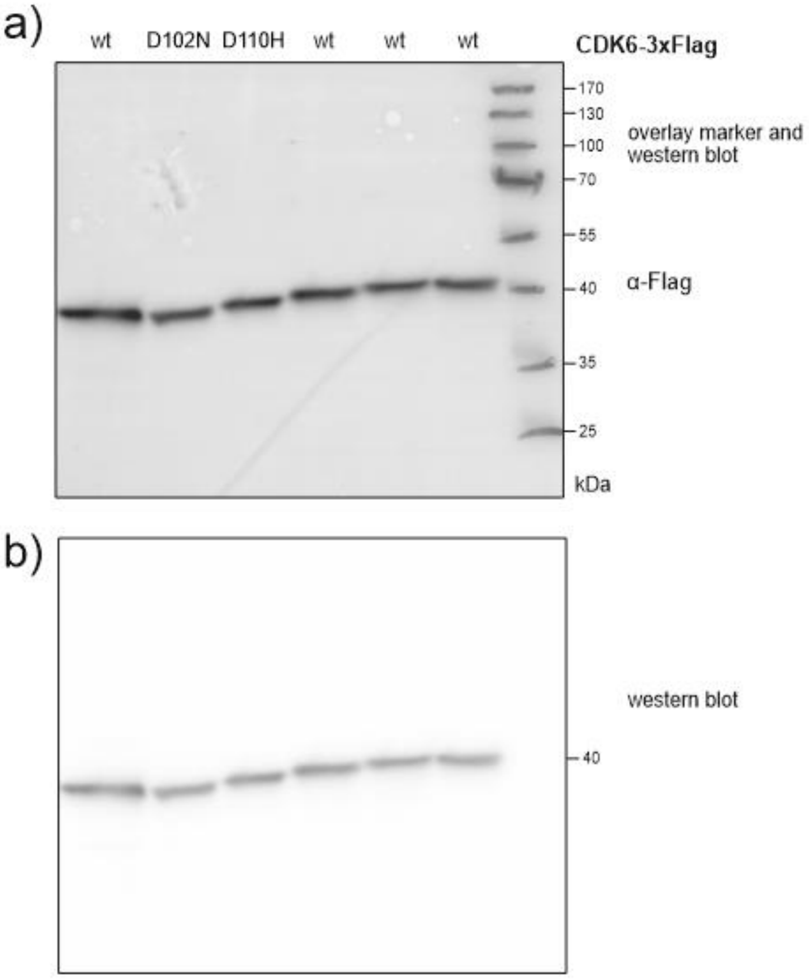

Supplementary Figure 2. a) Overlay of the used marker bands on the membrane with the detected western blot. b) Western blot of the whole uncut membrane is shown.
