# Supplementary material for: Disruptor: Computational identification of oncogenic mutants disrupting protein-protein and protein-DNA interactions
Source: Commun Biol. 2023 Jul 13;6:720. doi: 10.1038/s42003-023-05089-2 (PMC10344873; doi:10.1038/s42003-023-05089-2)
Supplement: Supplementary file 2 — Description of Additional Supplementary Data [file 42003_2023_5089_MOESM2_ESM.docx]

**Description of Additional Supplementary Files**

**File name:** Supplementary Data 1

**Description:** Exemplary input files for each interaction pair.

**File name:** Supplementary Data 2

**Description:** Osprey raw data and list of mutations predicted to disrupt binding for each interaction pair.

**File name:** Supplementary Data 3

**Description:** Source data for Figure 1e.
